# Idiopathic Intracranial Hypertension Managed With Lumboperitoneal Shunting and Venous Sinus Stenting: A Report of Two Cases

**DOI:** 10.7759/cureus.92771

**Published:** 2025-09-20

**Authors:** Yujiro Matsushima, Masato Saito, Takehiro Saga, Hajime Wada, Adam Tucker, Masao Sato, Manabu Kinoshita

**Affiliations:** 1 Neurological Surgery, Asahikawa Medical University, Asahikawa, JPN; 2 Neurosurgery, Asahikawa Medical University, Asahikawa, JPN; 3 Neurological Surgery, Hakodate Shintoshi Hospital, Hakodate, JPN; 4 Neurological Surgery, Japanese Red Cross Asahikawa Hospital, Asahikawa, JPN; 5 Neurological Surgery, Japanese Red Cross Kitami Hospital, Kitami, JPN; 6 Neurological Surgery, Sapporo Higashi Tokushukai Hospital, Sapporo, JPN

**Keywords:** cerebral venous sinus stenosis, idiopathic intracranial hypertension (iih), lumboperitoneal (lp) shunt, transverse sinus stenosis, venous sinus stenting (vss)

## Abstract

Idiopathic intracranial hypertension (IIH) is a relatively rare disease of unknown pathogenesis initially managed by conservative medical treatment. For intractable cases, there is a trend toward endovascular venous sinus stenting (VSS) over traditional CSF diversion by surgical shunting. However, currently, there is no clear consensus regarding the selection of the surgical approach. We describe two cases of IIH in young women, each presenting with severe headache and visual disturbances, both resistant to initial medical therapy, and both found to have different patterns of transverse sinus stenosis. The first patient was a 33-year-old woman who underwent lumboperitoneal shunt surgery for treatment of venous stenosis suspected to be caused secondarily by extrinsic compression from raised intracranial pressure, while the second patient was a 23-year-old woman who was treated by endovascular sinus stenting for a suspected intrinsic form of venous stenosis. Each patient resulted in complete symptom resolution after two years of follow-up. Based on the findings and analysis of these two representative cases, distinguishing the form of venous sinus stenosis may be helpful in the selection of a surgical approach.

## Introduction

Idiopathic intracranial hypertension (IIH) is a disease of unknown pathogenesis associated with intracranial hypertension typically found in young to middle-aged obese women [1]. This is in contrast to secondary intracranial hypertension due to conditions such as intracranial mass lesions (tumors, hemorrhage, infarction), and venous sinus thrombosis. Secondary causes can be excluded based on the medical history, imaging findings, and blood tests. Visual impairment is the key symptom, and surgical intervention is indicated when visual impairment progresses despite medical treatment. The recent increase in obesity in the overall population has been accompanied by a corresponding increase in reported cases of IIH [2]. Despite the establishment of IIH guidelines by the International Headache Society in 2023 [3], there is a lack of consensus regarding the optimal surgical approach for refractory cases. Although unilateral or bilateral transverse sinus stenosis is observed in many IIH patients, it remains unclear whether the stenosis is a cause or a consequence of increased intracranial pressure (ICP). This report describes two non-consecutive cases of IIH diagnosed in 2022 at different institutions with different forms of transverse sinus stenosis successfully treated by different methods, namely lumboperitoneal (LP) shunting and transverse sinus stenting.

## Case presentation

Case 1

**Figure 1:**
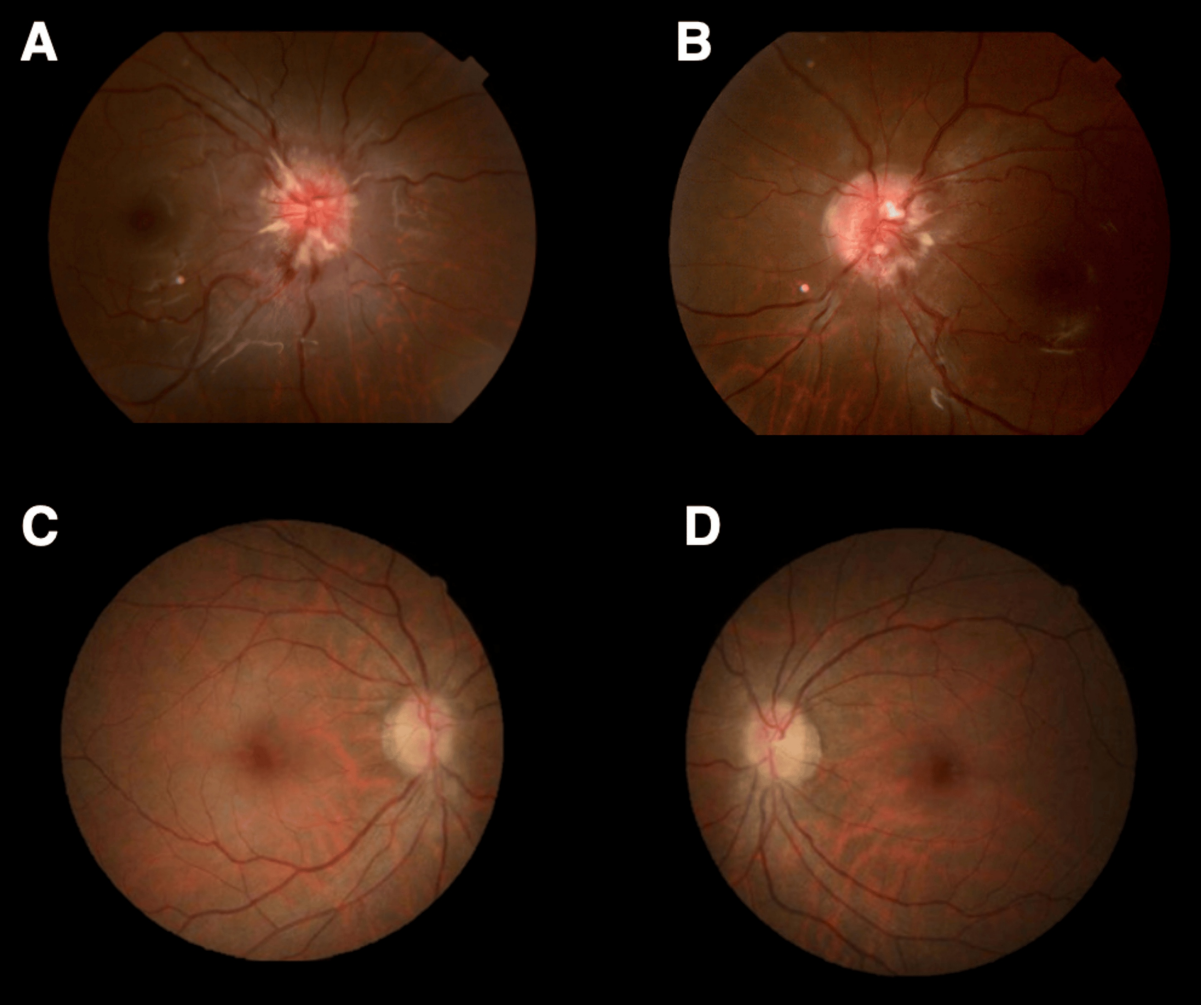
Pre- and postoperative retinal images of Case 1 Preoperative ophthalmoscopy shows bilateral papilledema (A: right eye, B: left eye). Postoperative ophthalmoscopy demonstrates decreased bilateral papilledema (C: right eye, D: left eye).

**Figure 2:**
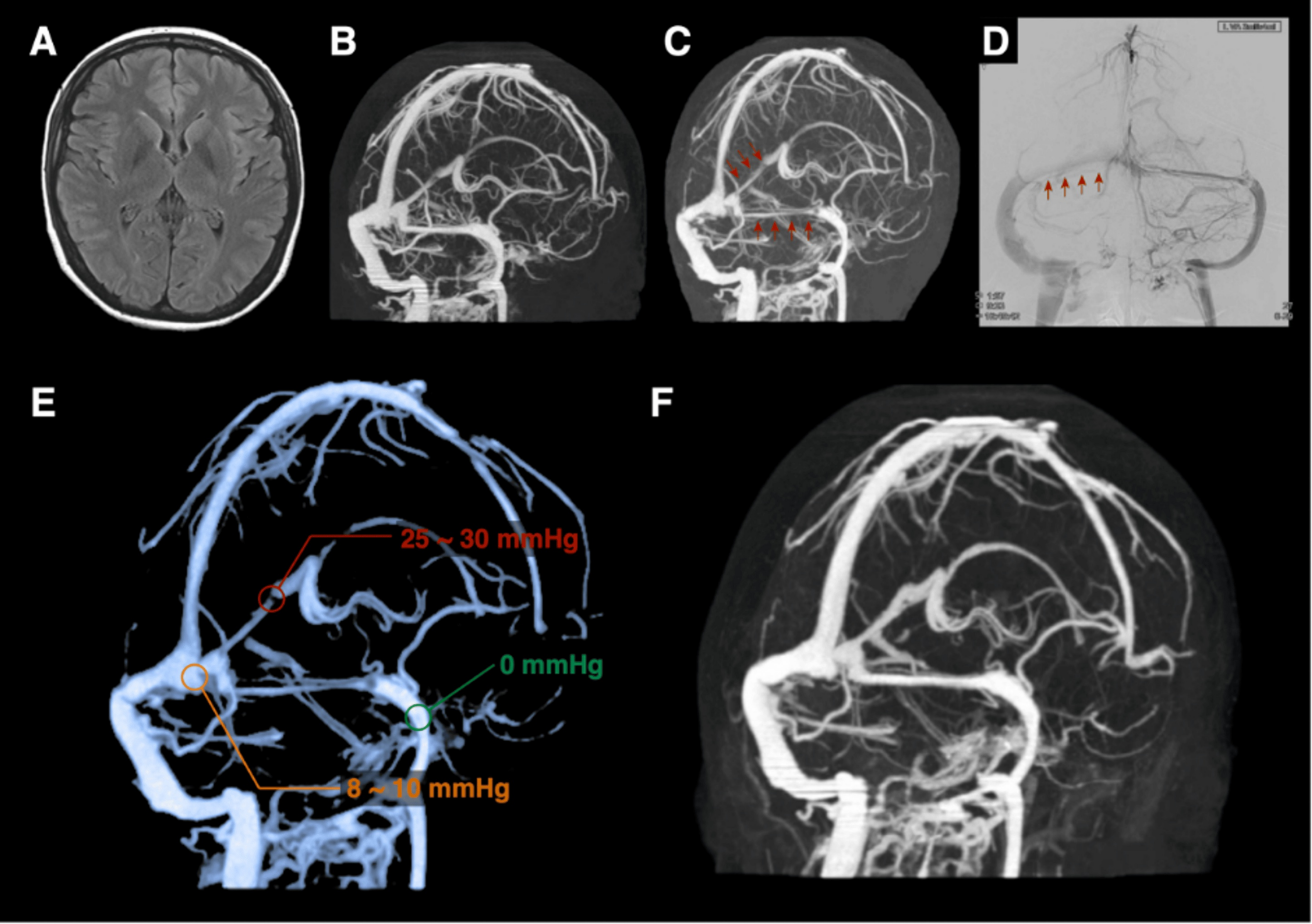
Pre- and postoperative images of Case 1 On admission, T2-weighted FLAIR and CTV images show no perisinus edema or other abnormalities (A, B). Follow-up CTV and DSA reveal venous sinus stenosis extending from the straight sinus to the right transverse sinus (C, D; red arrowheads indicate stenosis). CTV demonstrates pressure gradients at multiple locations (E). CTV performed one week after the LP shunt shows decreased sinus stenoses (F). FLAIR: fluid-attenuated inversion recovery; CTV: computed tomography venography; DSA: digital subtraction angiography; LP: lumboperitoneal

A 33-year-old woman with a body mass index (BMI) of 19.7 (height 167 cm, weight 55 kg) with no significant prior medical illnesses or history of oral contraceptive use presented to the Department of Neurology at our hospital with a progressive one-month history of intractable headache and blurry vision. Neurological exam was unremarkable, except for bilateral papilledema (Figures 1A-1B) and enlarged Mariotte blind spots. Laboratory studies showed no coagulation disorders, with a D-dimer of 0.9 μg/mL. Lumbar puncture demonstrated an elevated cerebrospinal fluid (CSF) pressure of 30 cmH_2_O. Radiological imaging, including magnetic resonance imaging (MRI), computed tomography angiography (CTA), and computed tomography venography (CTV), initially showed no abnormalities, with no findings of thrombus formation or perisinus edema (Figures 2A-2B). Four months later, symptoms worsened, and repeat MR venography (MRV) and CTV showed right transverse and straight venous sinus stenosis. The stenosis was non-localized with a uniformly narrowed diameter. Digital subtraction cerebral angiography (DSA) identified sinus stenosis at the same location (Figures 2C-2D). Venous sinus pressures measured by a microcatheter were 25-30 mmHg proximal to the stenosis at the straight sinus, 8-10 mmHg at the sinus commissure, and 0 mmHg at the left sigmoid sinus (Figure 2E). A diagnosis of IIH was suspected, and administration of acetazolamide failed to result in radiographic or clinical improvement. Considering that neurological symptoms preceded radiographic evidence of multiple venous sinus stenosis, it was suspected that the venous sinus stenosis was a result and not a cause of intracranial hypertension. Thus, after informed consent, an LP shunt (Strata valve, Medtronic, Minneapolis, MN, USA) was performed with an initial pressure setting of 7.0-8.5 cmH_2_O. The patient’s headache and visual blurring improved immediately after the operation, and CTV performed one week later showed resolution of the venous sinus stenosis at both the right transverse and straight sinus (Figure 2F). Neurological examination on discharge showed decreased papilledema (Figures 1C-1D), and at the two-year post-operative follow-up, there was no clinical or radiographic recurrence.

Case 2

**Figure 3:**
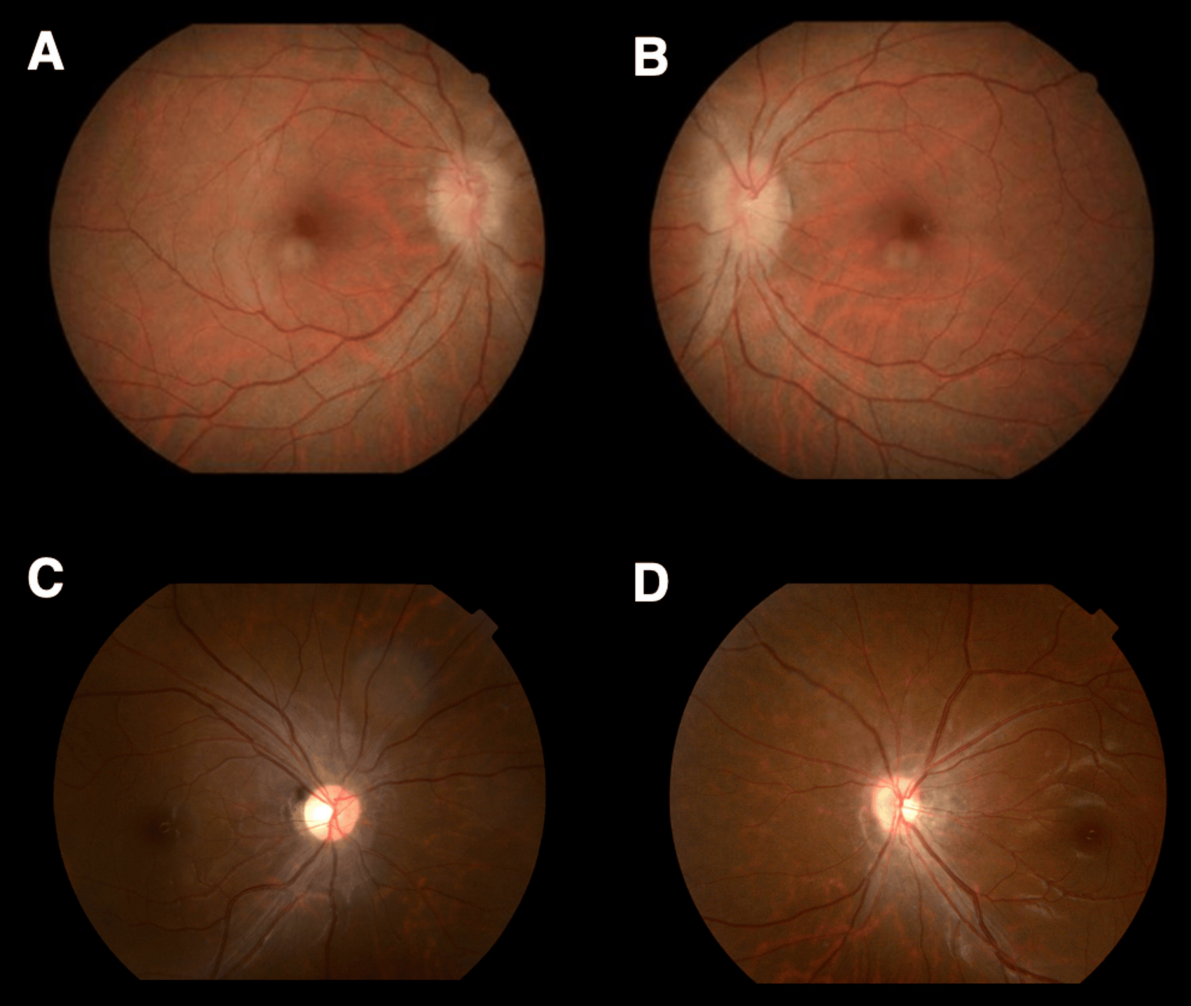
Pre- and postoperative retinal images of Case 2 Preoperative ophthalmoscopy shows bilateral papilledema (A: right eye, B: left eye). Postoperative ophthalmoscopy demonstrates decreased bilateral papilledema (C: right eye, D: left eye).

**Figure 4:**
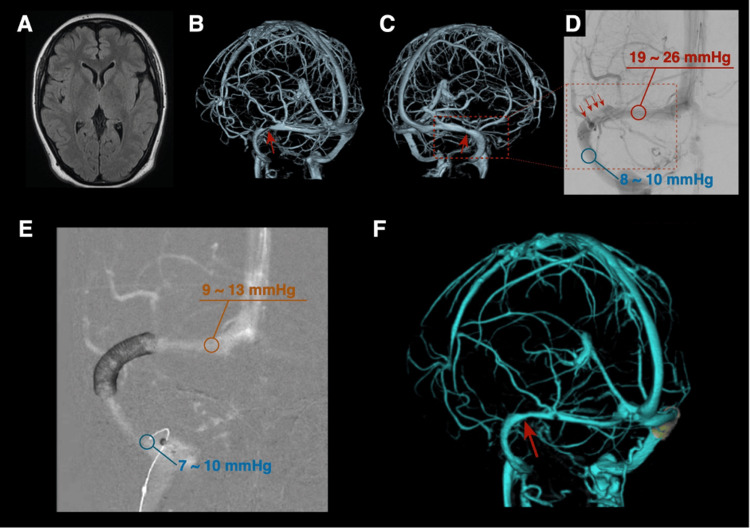
Pre- and postoperative images of Case 2 On admission, FLAIR imaging shows no perisinus edema or other abnormalities (A), while CTV demonstrates bilateral focal transverse sinus stenoses (B, C). DSA reveals right transverse sinus stenosis (red arrowheads) with a pressure gradient measured between the proximal and distal regions of the stenotic segment (D). Post-stent DSA shows placement across the stenotic lesion and a decreased venous pressure gradient immediately after stent deployment (E). CTV performed five days after treatment demonstrates decreased contralateral sinus stenosis. FLAIR: fluid-attenuated inversion recovery; CTV: computed tomography venography; DSA: digital subtraction angiography

A 23-year-old woman with a BMI of 25 (height 165 cm and weight 68 kg) and no prior medical illnesses or history of oral contraceptive use presented emergently to our hospital at the Department of Neurosurgery with a five-day history of headache, vomiting, and double vision. Neurological examination revealed bilateral abducens nerve palsies and bilateral papilledema (Figures 3A-3B). Laboratory tests showed no coagulation abnormalities, with D-dimer of 0.7 μg/mL. Lumbar puncture demonstrated an increased CSF pressure of 35 cmH_2_O. Although no abnormalities were observed on MRI (Figure 4A), CTV revealed localized bilateral transverse sinus stenoses (Figures 4B-4C). A diagnosis of IIH was suspected, and administration of acetazolamide, topiramate, and glycerin resulted in slight symptomatic improvement with no changes in papilledema. The right transverse sinus was dominant, and venous pressures measured using a guiding catheter placed distal and proximal to the stenosis were 8-10 mmHg and 19-26 mmHg, respectively (Figure 4D). Thus, this relatively high-pressure gradient confirmed the presence of a significant stenotic lesion. Based on these findings, and after informed consent, right transverse sinus stenting was performed. A carotid wall stent (8 x 29 mm; Boston Scientific, Natick, MA, USA) with prior off-label use approved by the treating institution was successfully deployed across the stenotic segment. Immediately after stent placement, venous pressure proximal to the right transverse sinus stenosis decreased to 9-13 mmHg (Figure 4E). CTV acquired on post-treatment day 5 showed improvement in not only the right transverse sinus stenotic lesion but also in the contralateral left transverse sinus stenosis (Figure 4F). On post-treatment day 6, the patient was asymptomatic, and the neurological exam revealed resolution of the abducens nerve palsy and decreased papilledema (Figures 3C-3D). There was no evidence of disease recurrence at the two-year post-operative follow-up examination.

## Discussion

Although various theories on the pathogenesis of IIH have been presented in the literature, currently the leading mechanisms include CSF overproduction or malabsorption [4-6]. However, the precise mechanism remains to be established, and possibly multiple factors are involved [7-9]. Because IIH is more commonly found in women of reproductive age, hormone disturbances have been postulated to be a factor in the pathogenesis [9]. Furthermore, obesity has been reported in up to 90% of IIH patients, and a 5-15% increase in body weight has been suggested to exacerbate symptoms [10]. Except for acute exacerbation, initial IIH management consists of weight control and medical treatment. Acetazolamide is the most commonly used oral medication for IIH, and the combined use of acetazolamide and weight loss is reported to be more effective than weight loss alone for improving visual acuity impairment [11]. In both of our cases, the BMI was within the normal range, suggesting the possibility of an underlying secondary cause of raised ICP, such as sinus thrombosis; however, this was excluded by laboratory and radiological studies.

Unilateral or bilateral transverse sinus stenosis has been observed in up to 90% of IIH patients [12]. There is no consensus on whether sinus stenosis precedes IIH as a cause or is a consequence of IIH. However, it has been postulated that increased ICP causes venous sinus stenosis at locations vulnerable to increased pressure, which leads to insufficient venous drainage, causing a vicious cycle that further increases ICP [3]. Case 1 is uniquely illustrative because increased ICP preceded the venous sinus stenosis, suggesting a possible mechanism whereby ICP may be a causal factor in vascular dynamics and vessel wall changes. Furthermore, this was a rare example of stenosis extending to the straight venous sinus that has not been reported in the recent literature. In Case 2, it is not clear whether the stenosis preceded or followed increased ICP.

Surgical treatment for IIH includes shunting, venous sinus stenting (VSS), and optic nerve sheath fenestration; however, there is no clear consensus regarding the selection of treatment strategy or optimal timing for intervention [13]. In recent years, the number of reports of stenting for IIH treatment has increased [14-17]. In stented IIH patients, 77.7% have been reported to experience symptom improvement, while 22.3% suffered persistent or worsened symptoms [17]. The total disease recurrence rate, including restenosis of the stent or the adjacent areas, is estimated as 17.7%, suggesting that sinus stenting alone is not a curative treatment. Further adjuvant therapy may be required, such as CSF shunting or optic nerve sheath fenestration [17].

CSF shunting is a treatment that results in a rapid decrease in ICP and is effective for decreasing papilledema and visual impairment [18]. However, there are also several drawbacks, such as infectious complications, shunt occlusion, shunt misplacement, and intracranial hypotension syndrome. These complications could require shunt reconstruction, further complicating the patient’s clinical condition. The recurrence rate of headaches treated by CSF shunting is estimated to be as high as 50% in a 36-month post-operative follow-up, and consequently, shunting is not recommended for patients with headache symptoms alone [19]. Although LP shunting or ventriculoperitoneal (VP) shunting are comparable treatment options, VP shunting is considered to have fewer complications and treatment failures, presumably due to relative familiarity with the technique; however, LP shunting, because of the lesser invasive nature, is frequently chosen by patients, as was in our case [19].

Our patients underwent different procedures, yet both showed remarkable responses to treatment with satisfactory two-year outcomes. The primary pathogenic mechanism in Case 1 was considered to be the underlying increased ICP, with venous sinus stenosis being a secondary phenomenon. In such cases, VSS may not be fundamentally curative, and recurrence may later arise. Furthermore, Case 1 uniquely shows the temporal progression of stenosis with worsening of symptoms. In most cases in the literature, venous sinus stenosis is found simultaneously with symptom onset; therefore, it is currently difficult to determine whether the stenosis is primary or secondary.

The significant difference between our two cases is the radiographic patterns of sinus stenosis. In Case 1, the sinus stenosis was uniform and relatively extensive, while the stenosis in Case 2 was more localized. A report on transverse sinus stenting for IIH has classified the venous sinus stenosis into two major morphological groups: either intrinsic stenosis (fibrous septae, arachnoid granulations) or extrinsic compression (primary increased ICP); however, stenting was performed in all cases, regardless of the type [20]. Based on this classification, Case 1 could be classified as extrinsic stenosis, while Case 2 could be classified as intrinsic. We believe this classification may be useful for determining the treatment method. Thus, for extrinsic stenosis, shunting would be indicated, while stenting would be more effective for intrinsic stenosis.

## Conclusions

Despite global trends favoring VSS for IIH, depending on the underlying pathophysiology, LP shunt may still be an appropriate option. The clinical course and radiological findings of our two representative cases highlight the possibility that intrinsic VSS may be the primary cause of elevated intracranial hypertension, and endovascular stenting would be appropriate. In contrast, in cases of extrinsic VSS, the stenosis may be a secondary consequence of elevated ICP, and CSF diversion by shunting may be more effective. Although the cause of IIH is most likely multifactorial, further studies on venous sinus morphology, CSF pressure dynamics, and long-term follow-up could enhance treatment decision-making, which could lead to better patient outcomes.
